# De novo serine synthesis regulates chondrocyte proliferation during bone development and repair

**DOI:** 10.1038/s41413-021-00185-7

**Published:** 2022-02-15

**Authors:** Steve Stegen, Shauni Loopmans, Ingrid Stockmans, Karen Moermans, Peter Carmeliet, Geert Carmeliet

**Affiliations:** 1 Laboratory of Clinical and Experimental Endocrinology, Department of Chronic Diseases and Metabolism, KU Leuven, 3000 Leuven, Belgium; 2 Laboratory of Angiogenesis and Vascular Metabolism, VIB Center for Cancer Biology, 3000 Leuven, Belgium; 3 Laboratory of Angiogenesis and Vascular Metabolism, Department of Oncology and Leuven Cancer Institute, KU Leuven, 3000 Leuven, Belgium; 4 State Key Laboratory of Ophthalmology, Zhongshan Ophthalmic Center, Sun Yat-Sen University, Guangzhou, 510080 China

**Keywords:** Homeostasis, Bone, Metabolomics

## Abstract

The majority of the mammalian skeleton is formed through endochondral ossification starting from a cartilaginous template. Cartilage cells, or chondrocytes, survive, proliferate and synthesize extracellular matrix in an avascular environment, but the metabolic requirements for these anabolic processes are not fully understood. Here, using metabolomics analysis and genetic in vivo models, we show that maintaining intracellular serine homeostasis is essential for chondrocyte function. De novo serine synthesis through phosphoglycerate dehydrogenase (PHGDH)-mediated glucose metabolism generates nucleotides that are necessary for chondrocyte proliferation and long bone growth. On the other hand, dietary serine is less crucial during endochondral bone formation, as serine-starved chondrocytes compensate by inducing PHGDH-mediated serine synthesis. Mechanistically, this metabolic flexibility requires ATF4, a transcriptional regulator of amino acid metabolism and stress responses. We demonstrate that both serine deprivation and PHGDH inactivation enhance ATF4 signaling to stimulate de novo serine synthesis and serine uptake, respectively, and thereby prevent intracellular serine depletion and chondrocyte dysfunction. A similar metabolic adaptability between serine uptake and de novo synthesis is observed in the cartilage callus during fracture repair. Together, the results of this study reveal a critical role for PHGDH-dependent serine synthesis in maintaining intracellular serine levels under physiological and serine-limited conditions, as adequate serine levels are necessary to support chondrocyte proliferation during endochondral ossification.

## Introduction

During endochondral bone formation, multipotent mesenchymal progenitors condense and differentiate into chondrocytes. Within this cartilaginous anlage, chondrocyte proliferation and hypertrophy control long bone growth, whereas the extracellular matrix they deposit is used as a template for bone formation by osteoblasts.^1^ These processes have to be strictly coordinated, because disruption of these events is often associated with the development of skeletal dysplasia.^2,3^ Moreover, since the cellular and molecular processes that contribute to bone regeneration mirror those occurring during skeletal growth, impaired chondrocyte function is also associated with delayed fracture healing or nonunion.^4,5^ Thus, understanding the molecular mechanisms that endow chondrocyte anabolism is necessary to accelerate the development of novel therapies for cartilage-related pathologies.

The regulation of chondrocyte differentiation and function by transcription factors and growth signals has long been established,^6,7^ but the metabolic control of these processes has not been fully elucidated. In contrast to most anabolic tissues, which are well vascularized, the developing growth plate and cartilaginous fracture callus do not contain blood vessels,^8^ suggesting that chondrocytes are characterized by a specific metabolic profile. Indeed, recent studies have shown that chondrocytes downregulate fatty acid uptake and oxidation^9^ and are therefore more dependent on other nutrients, such as glutamine and glucose, for their function. More specifically, glutamine-derived metabolites control the typical chondrogenic gene expression profile through epigenetic mechanisms and regulate biosynthesis and cell survival,^10^ whereas deletion of the main glucose transporter GLUT1 in chondrocytes reduces their proliferation and maturation but not their survival.^11^ Although the functional importance of glucose uptake in chondrocytes is evident, the metabolic fate of intracellular glucose is poorly characterized. The current understanding is that not only glycolysis but also glucose oxidation in the TCA cycle is necessary for ATP production.^12^ Whether glucose-derived carbon supports chondrocyte anabolism via other pathways is still not known.

A common trait in many anabolic (non)malignant cell types is de novo glucose-dependent serine synthesis even when sufficient extracellular serine is available.^13–16^ Initiated by the rate-limiting conversion of the glycolytic intermediate 3-phosphoglycerate (3PG) to 3-phosphohydroxypyruvate by phosphoglycerate dehydrogenase (PHGDH), the serine synthesis pathway (SSP) generates serine, which is subsequently used in several anabolic processes. For example, as a nonessential amino acid, serine directly fuels protein synthesis, but it is also incorporated in the head groups of certain lipids. Moreover, serine hydroxymethyltransferase (SHMT)-mediated conversion of serine into glycine charges the folate pool with the one-carbon units necessary for nucleotide synthesis.^13–16^ Finally, SSP-derived glycine also contributes to glutathione synthesis and thereby prevents the accumulation of harmful reactive oxygen species (ROS).^13–16^ Interestingly, transcriptome analysis has indicated that chondrocytes express PHGDH and other SSP-related enzymes,^17,18^ but whether and how de novo synthesized serine contributes to chondrocyte function is completely unknown.

Here, we investigate the functional role of the SSP in chondrocytes during endochondral ossification. We demonstrate that genetic deletion of PHGDH in growth plate chondrocytes impairs the synthesis of nucleotides necessary for proliferation, resulting in shortened limbs. In contrast, extracellular serine is less important for cartilage fitness, as serine-starved chondrocytes stimulate glucose-dependent serine synthesis through enhancement of activating transcription factor 4 (ATF4) signaling. Together, these data highlight the metabolic flexibility by which chondrocytes maintain an intracellular serine level necessary for their function during endochondral bone formation.

## Results

### De novo glucose-derived serine synthesis in chondrocytes

**Fig. 1:**
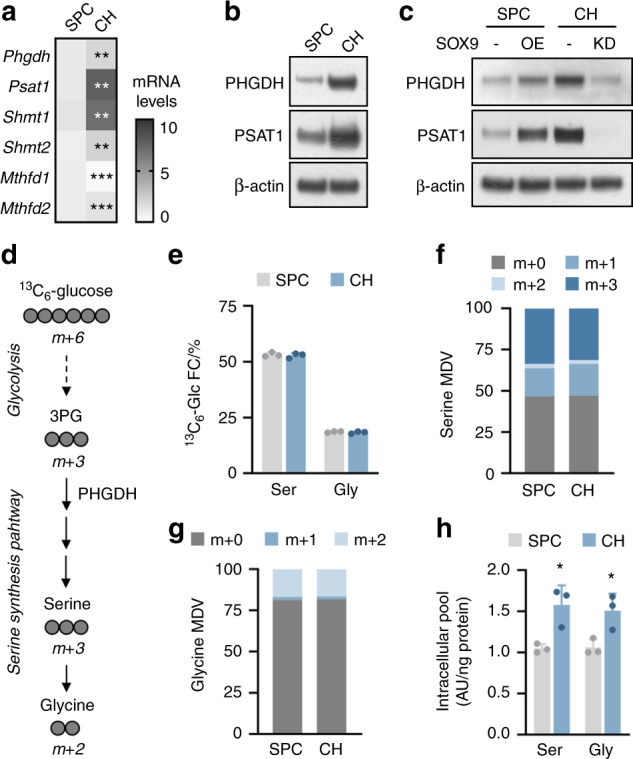
The serine synthesis pathway in growth plate chondrocytes. **a** *Phgdh*, *Psat1*, *Shmt1*, *Shmt2*, *Mthfd1*, and *Mthfd2* mRNA levels in cultured skeletal progenitor cells (SPCs) and growth plate chondrocytes (CHs) (*n* = 3). **b** Immunoblot of PHGDH, PSAT1 and β-actin in SPCs and CHs (*n* = 3). **c** Immunoblot of PHGDH, PSAT1 and β-actin in SPCs and CHs after transduction with a lentiviral vector carrying a SOX9 overexpression (OE) plasmid or a shRNA (KD) against SOX9 (*n* = 3). Empty vector or scrambled shRNA was used as the respective control. **d** Schematic of carbon atom (circles) transitions of ^13^C_6_-glucose used to detect label incorporation into the shown metabolites. **e** Fractional contribution (FC) of ^13^C_6_-glucose (^13^C_6_-Glc) to serine (Ser) and glycine (Gly) in SPCs and CHs (*n* = 3). Serine (**f**) and glycine (**g**) labeling from ^13^C_6_-glucose in SPCs and CHs (*n* = 3). Specific mass distribution vectors (MDVs) for each metabolite are shown. **h** Intracellular Ser and Gly levels in SPCs and CHs (*n* = 3). The data are presented as the means ± SDs; *P < 0.05 vs. SPCs, **P < 0.01 vs. SPCs, ***P < 0.001 vs. SPCs (Student’s *t* test)

We first assessed whether the SSP is an active metabolic pathway in chondrocytes. Compared to multipotent skeletal progenitor cells,^19^ growth plate chondrocytes showed higher PHGDH and phosphoserine aminotransferase 1 (PSAT1) mRNA and protein levels and higher gene expression levels of enzymes involved in one-carbon metabolism, such as SHMT1/2 and methylenetetrahydrofolate dehydrogenase 1/2 (MTHFD1/2) (Fig. 1a, b). The increased expression of SSP-related genes was linked to the chondrogenic phenotype, since genetic modulation of the expression of SOX9, the master chondrogenic transcription factor,^20^ altered the expression of SSP enzymes: SOX9 overexpression in skeletal progenitors increased PHGDH and PSAT1 levels, whereas deletion of SOX9 in lineage-committed chondrocytes reduced PHGDH and PSAT1 expression (Fig. 1c and Supplementary Fig. 1a). To further assess differences in de novo serine and glycine synthesis between these cell types, we performed ^13^C_6_-glucose tracing. Theoretically, uniformly labeled ^13^C-glucose is metabolized via 3PG to m + 3 serine (i.e., three ^13^C-labeled atoms) and subsequently to m + 2 glycine (i.e., two ^13^C-labeled atoms) (Fig. 1d). In both chondrocytes and skeletal progenitors, glucose-derived carbon contributed to a significant proportion of the total serine (~50% fractional contribution, with ~30% specifically m + 3) and glycine (~20% fractional contribution, with ~20% specifically m + 2) carbon pool (Fig. 1e–g); this contribution was higher than that in most (non)-malignant cell types.^21–23^ Interestingly, although the ^13^C incorporation pattern was similar in skeletal progenitors and chondrocytes, intracellular serine and glycine levels were significantly higher in chondrocytes, not because of more metabolite uptake but likely because of higher SSP flux (Fig. 1h and Supplementary Fig. 1b). Taken together, these data indicate that the SSP is an active metabolic pathway in growth plate chondrocytes.

### Deletion of chondrocytic PHGDH impairs longitudinal bone growth

**Fig. 2:**
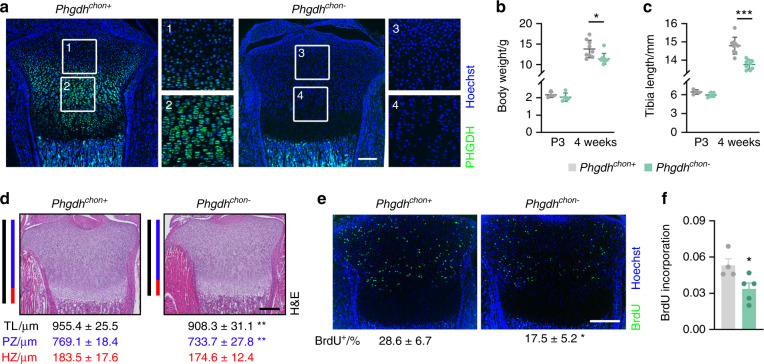
PHGDH in chondrocytes controls bone growth. **a** PHGDH immunostaining in neonatal growth plates from wild-type (*Phgdh*^*chon+*^) and chondrocyte-specific PHGDH knockout (*Phgdh*^*chon-*^) mice (*n* = 4–5). Enlarged images are shown for the boxed regions. Body weights (**b**) and tibia lengths (**c**) of mice at postnatal day 3 (P3) and at 4 weeks of age (*n* = 4–9). **d** H&E staining of the tibial growth plate at P3, with quantification of the total length (TL; black line) and the lengths of the proliferative zone (PZ; blue line) and hypertrophic zone (HZ; red line) (*n* = 4-5). **e** BrdU immunostaining in neonatal growth plates, with quantification of the percentage of BrdU-positive cells (*n* = 4-5). **f** Proliferation of cultured chondrocytes (*n* = 4–5), as determined by BrdU incorporation. The data are presented as the means ± SDs; *P < 0.05 vs. *Phgdh*^*chon+*^, **P < 0.01 vs. *Phgdh*^*chon+*^, ***P < 0.001 vs. *Phgdh*^*chon+*^ (Student’s *t* test). The scale bars in **a**, **d**, **e** represent 200 µm

To investigate the physiological role of PHGDH-mediated serine synthesis during endochondral bone development, we deleted PHGDH in growth plate chondrocytes by crossing *Phgdh*^*fl/fl*^ mice with *type 2 collagen* (*Col2*)-Cre transgenic mice (resulting in *Phgdh*^*chon−*^mice). This approach resulted in efficient and chondrocyte-specific deletion of PHGDH, as confirmed by qRT–PCR, Western blot analysis and immunohistochemistry (Fig. 2a and Supplementary Fig. 2a, b). At birth, *Phgdh*^*chon−*^ mice were viable and undistinguishable from their wild-type littermates, but their growth was progressively impaired, as evidenced by the decreases in body weight and tibia length (Fig. 2b, c). Although bone length was not affected in *Phgdh*^*chon−*^ mice at postnatal day 3, we observed a modest but significant decrease in the total length of the growth plate (Fig. 2d). The reduction in growth plate length in *Phgdh*^*chon−*^ mice suggested decreased chondrocyte proliferation, especially since the length of the proliferative zone but not the hypertrophic zone was decreased (Fig. 2d). Accordingly, the number of BrdU-positive proliferating cells was significantly reduced in *Phgdh*^*chon−*^ growth plates and cultured PHGDH-deficient chondrocytes (Fig. 2e, f). Of note, PHGDH deletion did not affect other properties of chondrocytes, such as extracellular matrix synthesis or cell survival (Supplementary Fig. 2c–g). In addition, trabecular and cortical bone parameters were not altered in *Phgdh*^*chon−*^ mice, as evidenced by microCT analysis of neonatal bones (Supplementary Fig. 2h–j). Together, these results indicate that chondrocytic PHGDH contributes to longitudinal bone growth during endochondral ossification by regulating proliferation.

### PHGDH regulates nucleotide synthesis in chondrocytes

**Fig. 3:**
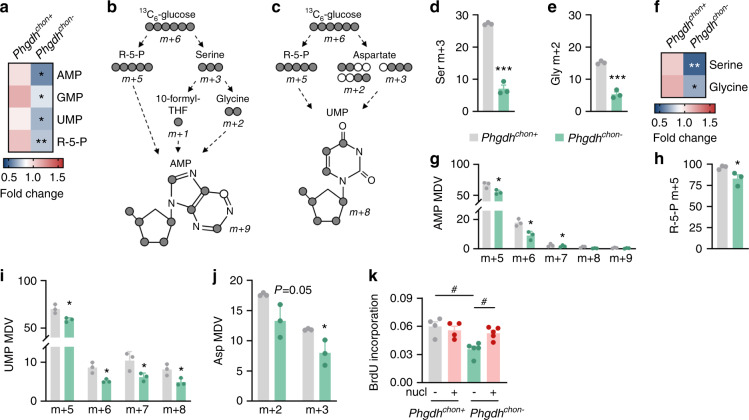
PHGDH regulates nucleotide synthesis in chondrocytes. **a** Purine (AMP, GMP), pyrimidine (UMP) and ribose-5-phosphate (R-5-P) levels in cultured chondrocytes derived from wild-type (*Phgdh*^*chon+*^) and chondrocyte-specific PHGDH knockout (*Phgdh*^*chon-*^) mice (*n* = 3). Schematic of carbon atom (circles) transitions of ^13^C_6_-glucose used to detect label incorporation into AMP (**b**) and UMP (**c**). Serine (Ser; **d**) and glycine (Gly; **e**) labeling from ^13^C_6_-glucose in cultured chondrocytes (*n* = 3). Specific mass distribution vectors (MDVs) for each metabolite are shown. **f** Intracellular Ser and Gly levels in cultured chondrocytes (*n* = 3). AMP (**g**), ribose-5-phosphate (R-5-P; **h**), UMP (**i**) and aspartate (Asp; **j**) labeling from ^13^C_6_-glucose in cultured chondrocytes (*n* = 3). Specific MDVs for each metabolite are shown. **k** Proliferation of cultured chondrocytes with or without nucleotide (nucl) supplementation (*n* = 4–5), as determined by BrdU incorporation. The data are presented as the means ± SDs; *P < 0.05 vs. *Phgdh*^*chon+*^, **P < 0.01 vs. *Phgdh*^*chon+*^, ***P < 0.001 vs. *Phgdh*^*chon+*^ (Student’s *t* test), ^#^*P* < 0.05 (ANOVA)

We next explored how inactivation of PHGDH impairs chondrocyte proliferation. Metabolically, proliferation greatly depends on cellular biosynthetic processes, such as nucleotide and protein synthesis, in addition to an adequate energy balance,^24,25^ and we therefore questioned whether these aspects are affected by PHGDH deletion. Protein synthesis and energy homeostasis were not altered in PHGDH-deficient chondrocytes (Supplementary Fig. 3a–c), whereas nucleotide metabolism was significantly impaired, as shown by mass spectrometry-based metabolic analysis. Indeed, we noted a reduction in purine (AMP and GMP) and pyrimidine (UMP) levels in mutant chondrocytes, accompanied by a decrease in ribose-5-phosphate (Fig. 3a), the pentose phosphate pathway (PPP) intermediate that serves as a scaffold for nucleotide synthesis. To further study how PHGDH controls nucleotide metabolism, we cultured chondrocytes in medium supplemented with ^13^C_6_-glucose and analyzed glucose carbon entry into pathways that support purine and pyrimidine synthesis (Fig. 3b, c). Glucose contributes to the purine precursor AMP through glycine and 10-formyltetrahydrofolate formation in the SSP and through ribose-5-phosphate synthesis in the PPP.^26^ As expected, PHGDH deletion reduced glucose-derived carbon incorporation into serine and glycine, thereby decreasing their levels, which resulted in less glucose-derived ^13^C incorporation into AMP (Fig. 3d–g). More specifically, the levels of m + 6 and m + 7 AMP isotopologues were decreased in PHGDH-null cells, indicating reduced labeling from serine-derived 10-formyltetrahydrofolate and glycine, respectively (Fig. 3g). The levels of the m + 5 isotopologues of AMP and ribose-5-phosphate were also significantly reduced (Fig. 3g, h), indicating that glucose-dependent ribose-5-phosphate synthesis via the PPP was decreased and therefore suggesting that PHGDH may also control nucleotide synthesis indirectly.^27^ We also noted a decrease in ^13^C incorporation from glucose into the pyrimidine precursor UMP (Fig. 3i), which, however, was not dependent on the incorporation of serine carbon. Theoretically, glucose can contribute carbon to UMP via PPP-derived ribose-5-phosphate and via aspartate derived from TCA cycle metabolites.^26^ Similar to the findings for AMP, the levels of m + 5 isotopologues of UMP were significantly decreased in PHGDH-deficient chondrocytes (Fig. 3i). Moreover, in line with previous reports,^27^ we found that glucose-derived carbon incorporation into TCA cycle intermediates was decreased, which resulted in a decreased amount of ^13^C-labeled aspartate, thereby explaining the reduction in m + 7 and m + 8 UMP isotopologues (Fig. 3j and Supplementary Fig. 3d–j). Thus, chondrocytic PHGDH controls nucleotide synthesis directly via de novo serine synthesis and indirectly by regulating glucose-derived carbon entry into the PPP and TCA cycle.

Finally, to functionally link the decrease in nucleotide synthesis with the defect in proliferation, we added a nucleoside mixture to cultured chondrocytes. While the proliferation of wild-type chondrocytes was not affected, nucleoside supplementation fully rescued the proliferation defect of PHGDH-deficient cells (Fig. 3k). Collectively, our data indicate that PHGDH-mediated nucleotide synthesis is essential for chondrocyte proliferation during endochondral ossification.

### Complementary action of serine uptake and synthesis in challenged chondrocytes

**Fig. 4:**
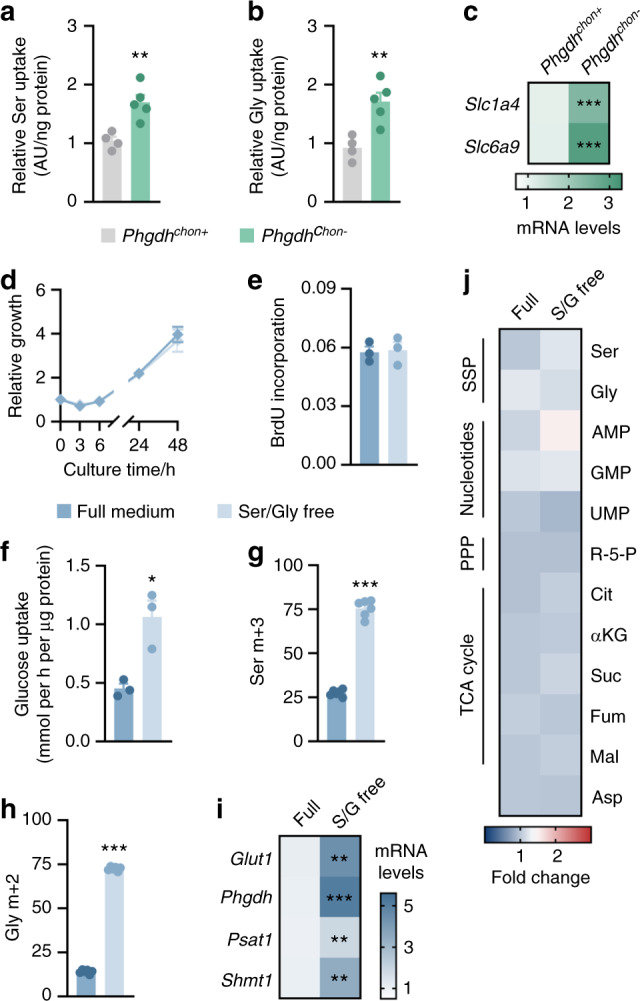
Complementary action of serine uptake and synthesis in challenged chondrocytes. Relative serine (Ser; **a**) and glycine (Gly; **b**) uptake in cultured chondrocytes derived from wild-type (*Phgdh*^*chon+*^) and chondrocyte-specific PHGDH knockout (*Phgdh*^*chon−*^) mice (*n* = 4–5). **c** *Slc1a4* and *Slc6a9* mRNA levels in wild-type and PHGDH-deficient chondrocytes (*n* = 3). **d** Growth curve of chondrocytes cultured in complete growth medium or serine/glycine-free medium (*n* = 3), based on their DNA content. **e** Proliferation of chondrocytes cultured in complete growth medium or serine/glycine-free medium (*n* = 3), as evidenced by BrdU incorporation. **f** Glucose uptake in chondrocytes cultured in complete growth medium or serine/glycine-free medium (*n* = 4–5). Serine (**g**) and glycine (**h**) labeling from ^13^C_6_-glucose in chondrocytes cultured in complete growth medium or serine/glycine-free medium (*n* = 6). Specific mass distribution vectors for each metabolite are shown. **i** *Glut1*, *Phgdh*, *Psat1*, and *Shmt1* mRNA levels in chondrocytes cultured in complete growth medium or serine/glycine-free medium (*n* = 3). **j** Intracellular levels of the indicated metabolites in chondrocytes cultured in complete growth medium or serine/glycine-free medium (*n* = 4-5). R-5-P represents ribose-5-phosphate, Cit represents citrate, αKG represents α-ketoglutarate, Suc represents succinate, Fum represents fumarate, Mal represents malate and Asp represents aspartate. The data are presented as the means ± SDs; *P < 0.05 vs. *Phgdh*^*chon+*^, **P < 0.01 vs. *Phgdh*^*chon+*^, ***P < 0.001 vs. *Phgdh*^*chon+*^ (Student’s *t* test)

Despite the fact that chondrocytes rely on de novo serine synthesis for nucleotide synthesis, we noted that a significant portion of the intracellular serine and glycine carbon pool in growth plate chondrocytes was not labeled by glucose-derived carbon (Fig. 1f, g), which prompted us to investigate the functional importance of exogenous serine and glycine. Chondrocytes indeed take up a significant portion of the serine and glycine in culture medium, and this amount was found to be further increased upon inactivation of PHGDH (Fig. 4a, b). This effect correlated with the enhanced mRNA expression of *Slc1a4* and *Slc6a9* (Fig. 4c), which encode amino acid transporters that mediate serine and glycine uptake. Consistent with this finding, we observed a similar increase in *Phgdh*^*chon-*^ growth plates (*Slc1a4*: 1.9-fold increase, *P* = 0.012; *Slc6a9*: 2.7-fold increase, *P* = 0.002). Thus, to prevent complete depletion of intracellular serine and glycine, PHGDH knockout chondrocytes partially compensate by increasing their serine and glycine uptake.

To further investigate whether serine availability affects chondrocyte function, we cultured wild-type cells in serine/glycine-free medium. Glycine was also removed from the medium because glycine and serine can be interconverted, and this approach ensured that cells were not able to synthesize serine from exogenous glycine. Surprisingly, combined serine and glycine withdrawal did not impair chondrocyte proliferation (Fig. 4d, e), nor did it affect other cellular functions, such as survival and extracellular matrix deposition (Supplementary Fig. 4a, b). We reasoned that serine/glycine-deprived chondrocytes enhanced their glucose-dependent serine production to maintain the intracellular serine pools necessary for cell proliferation. Indeed, culturing chondrocytes in serine/glycine-free medium enhanced the uptake of glucose and its conversion to serine and glycine, as demonstrated by ^13^C_6_-glucose tracing and gene expression analysis (Fig. 4f–i). As a result, the levels of SSP and TCA cycle intermediates and nucleotides were comparable between serine/glycine-starved chondrocytes and cells cultured in complete medium (Fig. 4j). The importance of glucose catabolism for de novo serine synthesis was further underscored by culturing chondrocytes in low glucose-containing medium. While decreasing only the glucose level by 5-fold did not noticeably affect chondrocyte function, simultaneous deprivation of glucose, serine and glycine impaired proliferation, survival and matrix deposition (Supplementary Fig. 4c–f).

**Fig. 5:**
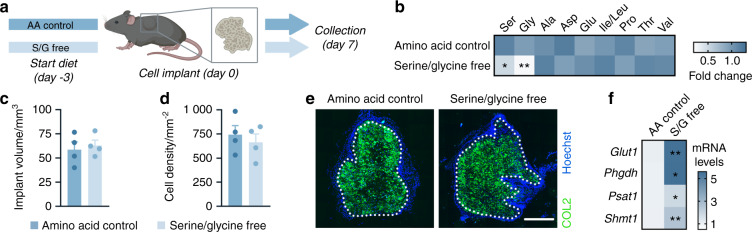
Serine/glycine-free diet feeding does not affect chondrocyte function in vivo. **a** Schematic overview of the experimental setup. Chondrogenically primed progenitor cells were ectopically implanted into mice fed an amino acid (AA) control diet or a serine/glycine (S/G)-free diet. Ossicles were collected 7 days after implantation. **b** Serum metabolite levels in mice fed the control or serine/glycine-free diet (*n* = 4). Ser represents serine, Gly represents glycine, Ala represents alanine, Asp represents aspartate, Glu represents glutamate, Ile represents isoleucine, Leu represents leucine, Pro represents proline, Thr represents threonine and Val represents valine. Volume (**c**) and cell density (**d**) of ectopic implants (*n* = 4). **e** Type 2 collagen (COL2) immunostaining in ectopic implants (*n* = 4). The scale bar represents 500 µm. **f** *Glut1*, *Phgdh*, *Psat1*, and *Shmt1* mRNA levels in ectopic implants (*n* = 4). The data are presented as the means ± SDs; *P < 0.05 vs. AA control, **P < 0.01 vs. AA control (Student’s *t* test)

To confirm that chondrocytes can compensate for low extracellular serine levels by activating the SSP in vivo, we analyzed bone formation in an engineered ossicle implanted in mice fed a serine/glycine-free diet (Fig. 5a). The advantage of using this ectopic model is that it mimics endochondral bone development, as evidenced by the initial deposition of a COL2-positive cartilaginous matrix that is later replaced by bone tissue.^28^ Feeding mice a serine/glycine-free diet stably lowered their serum plasma levels by at least 2-fold without affecting the levels of other (nonessential) amino acids (Fig. 5b), suggesting that overall amino acid homeostasis was not altered. In accordance with our in vitro observations, withdrawal of dietary serine and glycine did not affect chondrocyte behavior in ectopic ossicles, likely because PHGDH-dependent serine production was induced (Fig. 5c–f). Thus, wild-type chondrocytes can withstand serine and glycine starvation by upregulating de novo serine synthesis.

### ATF4 regulates serine metabolism in chondrocytes

**Fig. 6:**
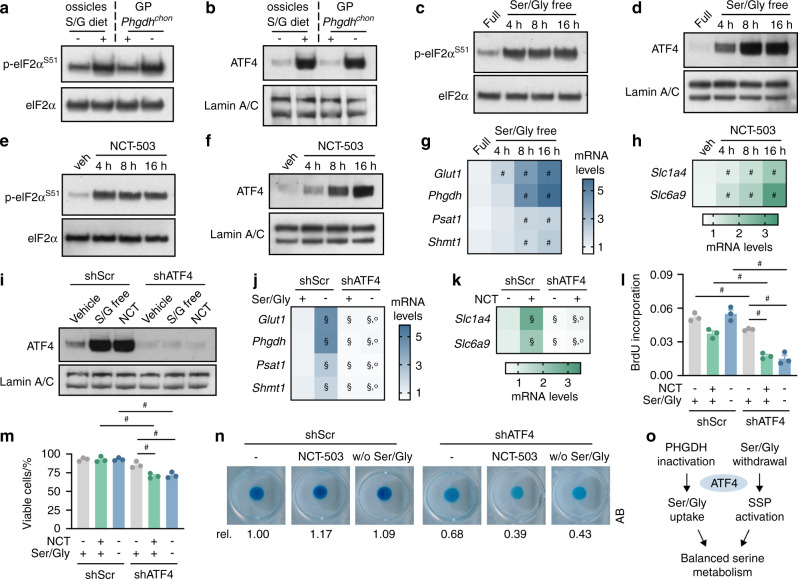
ATF4 mediates the cellular response to serine metabolic challenge. Immunoblots of phosphorylated eIF2α (Serine 51; p-eIF2α^S51^) and eIF2α (**a**) and of nuclear ATF4 and Lamin A/C (**b**) in bone ossicles and growth plates (GPs). For ectopic implants, chondrogenically primed wild-type progenitors were used as described in Fig. 5a and implanted in mice fed a normal chow (−) or serine/glycine (S/G)-free diet (+). Growth plates were dissected from wild-type (*Phgdh*^*chon+*^) and chondrocyte-specific PHGDH knockout (*Phgdh*^*chon−*^) mice (*n* = 3). Immunoblots of p-eIF2α^S51^ and eIF2α (**c**) and of nuclear ATF4 and Lamin A/C (**d**) in chondrocytes cultured in serine/glycine (Ser/Gly)-free medium for the indicated durations (*n* = 3). Immunoblots of p-eIF2α^S51^ and eIF2α (**e**) and of nuclear ATF4 and Lamin A/C (**f**) in chondrocytes treated with vehicle (veh) or NCT-503 for the indicated durations (*n* = 3). **g** *Glut1*, *Phgdh*, *Psat1*, and *Shmt1* mRNA levels in chondrocytes cultured in Ser/Gly-free medium for the indicated durations (*n* = 3). **h** *Slc1a4* and *Slc6a9* mRNA levels in chondrocytes treated with NCT-503 for the indicated durations (*n* = 3). **i** Immunoblot of nuclear ATF4 and Lamin A/C in chondrocytes after transduction with a lentiviral vector carrying a shRNA against ATF4 (*n* = 3). A scrambled shRNA (shScr) was used as the control. Control and ATF4-knockout cells were cultured in serine/glycine-free medium or treated with NCT-503 (NCT). **j** *Glut1*, *Phgdh*, *Psat1*, and *Shmt1* mRNA levels in control and ATF4-deficient chondrocytes cultured in complete medium (+) or serine/glycine-free medium (−) (*n* = 3). **k** *Slc1a4* and *Slc6a9* mRNA levels in control and ATF4-deficient chondrocytes treated with or without NCT-503 (*n* = 3). NCT- represents vehicle-treated. **l** Proliferation of control and ATF4-deficient chondrocytes cultured in complete or serine/glycine-free medium or treated with or without NCT-503 (*n* = 3), as evidenced by BrdU incorporation. NCT- represents vehicle-treated. **m** Viability of control and ATF4-deficient chondrocytes cultured in complete or serine/glycine-free medium or treated with or without NCT-503 (*n* = 3), as determined by Annexin V (AnxV)-propidium iodide (PI) staining followed by flow cytometry. AnxV^-^PI^-^ cells were considered viable. NCT- represents vehicle-treated. **n** Matrix deposition of control and ATF4-deficient chondrocytes cultured in complete or serine/glycine-free medium or treated with or without NCT-503 (*n* = 3), as evidenced by Alcian Blue (AB) staining. NCT represents vehicle-treated. **o** Schematic overview of the role of ATF4 in the regulation of serine metabolism in chondrocytes. The data are presented as the means ± SDs; ^#^*P* < 0.05 (ANOVA), ^§^*P* < 0.05 vs. shScr-complete medium or shScr-vehicle (ANOVA), °*P* < 0.05 vs. shScr-Ser/Gly-free or shScr-NCT (ANOVA)

We next explored the molecular mechanism by which chondrocytes cope with impaired serine metabolism caused by either PHGDH inactivation or serine/glycine deprivation. In response to amino acid stress, cells generally activate the eukaryotic initiation factor 2α (eIF2α)-ATF4 signaling pathway. When intracellular serine levels are low, phosphorylation of eIF2α results in ATF4-dependent transactivation of genes involved in the SSP or uptake of serine and glycine, depending on whether exogenous serine is available.^29–32^ In ossicles derived from mice fed a serine/glycine-free diet and in growth plates isolated from *Phgdh*^*chon-*^ mice, we found that the phosphorylated eIF2α and nuclear ATF4 levels were significantly increased (Fig. 6a, b). In accordance with this finding, serine/glycine deprivation or pharmacological PHGDH inhibition in cultured chondrocytes rapidly induced eIF2α-ATF4 signaling, accompanied by increased mRNA levels of SSP enzymes or transporters that facilitate serine and glycine uptake (Fig. 6c–h). Of note, pharmacological PHGDH inhibition functionally mirrored gene inactivation completely (Supplementary Fig. 5a–h). Thus, these findings indicate that SSP inactivation or serine/glycine unavailability results in activation of eIF2α-ATF4 signaling, which in turn induces the expression of genes involved in serine/glycine uptake or de novo serine synthesis, respectively.

To confirm the functional importance of the ATF4 pathway in the adaptation of serine metabolism, we cultured chondrocytes expressing scrambled shRNA or shATF4 in serine/glycine-free medium or in the presence of the PHGDH inhibitor NCT-503. ATF4 deletion completely blocked the compensatory increase in gene expression upon serine/glycine withdrawal or PHGDH inhibition and resulted in reduced proliferation, cell survival and matrix deposition (Fig. 6i–n). Taken together, these data indicate that ATF4-dependent regulation of serine metabolism is critical for chondrocyte function (Fig. 6o).

### Simultaneous impairment of de novo serine synthesis and serine uptake results in severe functional defects

**Fig. 7:**
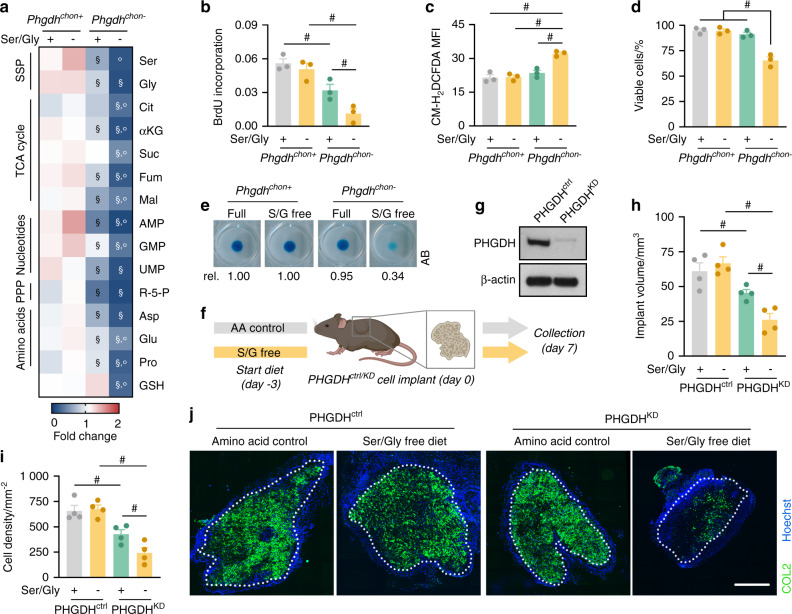
Combined deficiency of serine uptake and de novo serine synthesis severely affect chondrocyte function. **a** Intracellular levels of the indicated metabolites in chondrocytes isolated from wild-type (*Phgdh*^*chon+*^) and chondrocyte-specific PHGDH knockout (*Phgdh*^*chon−*^) mice and cultured in complete growth medium (+) or serine/glycine-free medium (−) (*n* = 3). Ser represents serine, Gly represents glycine, Cit represents citrate, αKG represents α-ketoglutarate, Suc represents succinate, Fum represents fumarate, Mal represents malate, R-5-P represents ribose-5-phosphate, Asp represents aspartate, Glu represents glutamate, Pro represents proline and GSH represents reduced glutathione. Proliferation (**b**; quantified by BrdU incorporation), ROS content (**c**; quantified by the CM-H_2_DCFDA mean fluorescence intensity, MFI), viability (**d**; quantified by AnxV-PI flow cytometry, viable cells are AnxV^-^PI^-^) and matrix deposition (**e**; Alcian Blue (AB) staining) of wild-type and PHGDH-deficient chondrocytes cultured in complete (+) or serine/glycine (S/G)-free medium (−) (*n* = 3). **f** Schematic overview of the experimental setup. Chondrogenically primed control (PHGDH^ctrl^) or PHGDH-deficient (PHGDH^KD^) progenitor cells were ectopically implanted into mice fed an amino acid (AA) control diet or a serine/glycine-free diet. Ossicles were collected 7 days after implantation. **g** Immunoblot of PHGDH and β-actin in PHGDH^ctrl^ and PHGDH^KD^ cells prior to implantation (*n* = 4). Volume (**h**) and cell density (**i**) of PHGDH^ctrl^ and PHGDH^KD^ ectopic implants (*n* = 4). **j** COL2 immunostaining in PHGDH^ctrl^ and PHGDH^KD^ ectopic implants (*n* = 4). The scale bar represents 500 µm. The data are presented as the means ± SDs; ^§^*P* < 0.05 vs. *Phgdh*^*chon+*^-complete medium, °*P* < 0.05 vs. *Phgdh*^*chon−*^complete medium, ^#^*P* < 0.05 (ANOVA)

The metabolic flexibility that allows chondrocytes to cope with dysregulated serine metabolism suggests that maintaining intracellular serine/glycine levels is critical for chondrocyte function. To test this hypothesis, we simultaneously blocked the SSP and reduced serine/glycine availability in the diet. Culturing PHGDH-deficient chondrocytes in serine/glycine-free medium almost completely depleted intracellular serine and glycine and profoundly decreased the levels of metabolic intermediates of the TCA cycle and the PPP (Fig. 7a). As expected, purine and pyrimidine levels were significantly decreased, which resulted in a pronounced reduction in proliferation compared to that of knockout cells cultured in full medium (Fig. 7a, b). Combined PHGDH inactivation and serine/glycine starvation also disrupted other cellular functions, which were not affected by either enzyme or nutrient deficiency alone. First, we observed a decrease in reduced glutathione (GSH) levels, which was likely caused by the decreases in the amino acids glutamate and glycine, precursors of GSH (Fig. 7a). In turn, ROS levels were increased, which resulted in cell death (Fig. 7c, d). Second, matrix deposition in micromass cultures was impaired when PHGDH-deficient chondrocytes were cultured in serine/glycine-free medium, consistent with the decreases in amino acids necessary for protein and collagen synthesis (Fig. 7a, e).^33^ Taken together, our in vitro data indicate that maintaining intracellular serine homeostasis is critical for chondrocyte function.

Finally, we investigated the importance of exogenous serine availability for PHGDH-deficient chondrocytes in vivo using two models. In the ectopic bone ossicle model, chondrogenically primed control (PHGDH^ctrl^) or PHGDH-deficient (PHGDH^KD^) progenitor cells were implanted in mice fed either normal chow or a serine/glycine-free diet (Fig. 7f, g). Ossicle formation by PHGDH^ctrl^ cells was not affected in serine-starved mice (Fig. 7h–j), likely because of compensatory enhancement of the SSP (Fig. 5f). In contrast, we found that PHGDH^KD^ ossicles from mice fed the control diet were smaller than PHGDH^ctrl^ ossicles, as evidenced by their lower cell density, whereas the percentage of COL2-positive matrix was not affected (Fig. 7h–j). This observation was in line with the proliferation defect observed in *Phgdh*^*chon-*^ growth plates (Fig. 2e). Moreover, when PHGDH^KD^ progenitor cells were implanted in mice fed the serine/glycine-free diet, ossicle volume, cell density and COL2 deposition were markedly decreased (Fig. 7h–j), thereby mirroring our in vitro observations that intracellular serine depletion strongly impairs chondrocyte function.

In the second model, we investigated whether serine metabolism also contributes to endochondral fracture healing (Supplementary Fig. 6a), which reiterates the sequence of events occurring during development. Similar to the observations in the ossicle model, the callus size and deposition of COL2-positive matrix were not altered in mice fed the serine/glycine-free diet (Supplementary Fig. 6b–d), likely because of activation of de novo serine synthesis. In contrast, local injection of the PHGDH inhibitor NCT-503 into a fracture callus reduced the callus size without affecting the amount of COL2-positive cartilage. When mice were fed the serine/glycine-free diet, the calluses were smaller and contained less COL2 upon cotreatment with NCT-503 (Supplementary Fig. 6b–d), indicating that maintaining intracellular serine levels is also important during fracture repair.

Collectively, these data argue that a major effect of the high PHGDH-mediated serine synthesis in chondrocytes is to buffer fluctuations in serine availability, thereby protecting these cells from the detrimental effects of intracellular serine depletion.

## Discussion

Highly anabolic chondrocytes survive and function in an avascular environment,^6–8^ suggesting that these cells are characterized by a specific metabolic profile. In addition to glutamine, glucose is a vital nutrient for chondrocyte function,^10–12^ but the metabolic fate of intracellular glucose is not yet fully understood. Using isotopic labeling and in vivo genetic models, we here show that the SSP supports the synthesis of nucleotides necessary for chondrocyte proliferation during endochondral bone formation and can compensate for low extracellular serine levels.

Skeletal growth and repair through endochondral ossification depends critically on rapidly proliferating chondrocytes that subsequently undergo hypertrophy.^4,34^ Adequate proliferation is generally supported by sufficient generation of nucleotides and amino acids while a proper energy balance is maintained,^24,25^ but the nutritional control of this anabolic process in chondrocytes is not yet fully understood. We recently showed that chondrocyte proliferation depends on the metabolism of glutamine via transaminases to generate nucleotides and amino acids.^10^ We now expand this mechanistic model by showing that glucose-dependent serine synthesis also supports chondrocyte proliferation by regulating nucleotide metabolism through simultaneous effects on several metabolic pathways. First, and consistent with the established role of the SSP in purine synthesis,^13–16^ inactivation of PHGDH decreased the intracellular AMP and GMP pool via reduced glycine carbon incorporation and one-carbon metabolism. Second, the intracellular levels of aspartate, a metabolic precursor for pyrimidine synthesis,^26^ were decreased in mutant chondrocytes, likely because of impaired transaminase activity. Indeed, the conversion of glucose into serine involves PSAT1 transaminase activity, through which glutamate is simultaneously converted into αKG, a critical TCA cycle intermediate that indirectly supports nucleotide synthesis through aspartate synthesis.^10,22^ In PHGDH-deficient chondrocytes, we found that the relative decrease in ^13^C-glucose labeling of αKG (−25%) could not account for the 40% reduction in the αKG level, indicating decreased conversion of glutamate to αKG, likely via PSAT1, and consequently decreased fueling of the TCA cycle and generation of aspartate. Third, using ^13^C-glucose tracing, we also discovered a role for PHGDH in regulating glucose carbon flux through the PPP, thereby indirectly affecting nucleotide synthesis via decreased generation of ribose-5-phosphate.^26^ Together, these observations suggest a mechanism by which the SSP is coupled to—and thus controls—several metabolic pathways that fuel chondrocyte proliferation, similar to observations in certain tumor cell lines.^27^

Our data indicate that compared to other cell types, chondrocytes are highly flexible in serine synthesis and utilization. First, in contrast to certain tumor cells,^35^ chondrocytes tolerate serine starvation well, even though almost 50% of the intracellular serine pool is derived from uptake. Mechanistically, serine-starved chondrocytes compensate by rapidly inducing eIF2α-ATF4 signaling, which in turn increases the expression of SSP-related enzymes to maintain intracellular serine homeostasis and thereby prevents cellular dysfunction. ATF4-dependent activation of the SSP in chondrocytes also appears to be physiologically relevant, since the proliferative zone in growth plates from ATF4^−/−^ neonatal mice is smaller and disorganized,^36^ suggesting that impaired SSP-dependent nucleotide synthesis might be at least a partial contributing factor. Second, in contrast to other anabolic cell types,^13–16^ PHGDH-deficient chondrocytes did not display changes in protein synthesis or redox homeostasis. A possible explanation is that deletion of PHGDH does not completely deplete the intracellular serine pool, likely because chondrocytes partially compensate by increasing serine uptake through ATF4 activation. Accordingly, PHGDH inactivation in combination with serine starvation completely depletes the intracellular serine pool, resulting in a more severe phenotype characterized by impaired biosynthesis accompanied by disrupted redox homeostasis and increased cell death.

Collectively, our data suggest that a critical role of the SSP is to protect chondrocytes against fluctuations in serine availability, and we speculate that this metabolic flexibility is especially relevant during fracture healing, when damage to the surrounding vasculature hinders a proper nutrient supply.^37^ We recently showed that during bone repair, low lipid availability promotes chondrogenesis^9^ and that this phenotypic switch is beneficial for survival and function in an avascular environment. Indeed, SOX9-driven induction of chondrogenesis is linked to a specific metabolic profile characterized by a low fatty acid oxidation rate, enhanced glycolysis and high glutamine catabolism.^9,10^ Based on our present study, we speculate that the high glycolytic flux in chondrocytes, in addition to energy generation, permits glucose-derived carbon flux through other anabolic pathways, such as the SSP, to sustain the high biosynthetic needs of these cells. Intriguingly, PHGDH expression was highest in chondrocytes localized in the center of the prehypertrophic and hypertrophic zones, which are the regions with the lowest oxygen levels,^8^ suggesting that activation of the hypoxia signaling pathway induces SSP-mediated serine synthesis to sustain chondrocyte proliferation under oxygen- and nutrient-scarce conditions. Whether and how other metabolic pathways adapt to changes in nutrient and oxygen availability and thereby regulate chondrocyte fate and function requires further study.

Taken together, our findings provide new insights into the regulatory role of glucose in chondrocyte function. We propose that glucose, through PHGDH-mediated metabolism, is necessary for chondrocyte proliferation by sustaining nucleotide synthesis and thereby controls bone growth during endochondral ossification. Moreover, chondrocytes are protected from serine starvation by upregulating de novo serine synthesis through increased ATF4 signaling, suggesting that the metabolic flexibility by which chondrocytes adapt to nutrient availability might be beneficial under conditions of acute nutrient shortage, as observed during fracture repair.

## Materials and methods

### Animals

Mice with chondrocyte-specific deletion of PHGDH were obtained by crossing *Phgdh*^*fl/fl*^ mice (in which *Phgdh* exons 4 and 5 are flanked by LoxP sites^38^) with *type 2 collagen* gene promoter-Cre (*Col2*-Cre) transgenic mice^39^ (resulting in *Col2*-Cre^+^;*Phgdh*^*fl/fl*^ mice, referred to herein as *Phgdh*^*chon−*^ mice). *Col2*-Cre*−*;*Phgdh*^*fl/fl*^ (*Phgdh*^*chon+*^) littermates were used as controls in all experiments. Mouse phenotyping was performed on male mice at postnatal day 3 unless mentioned otherwise. Mice (100% C57BL/6J background) were housed and bred under conventional conditions (Proefdierencentrum Leuven, Belgium), and approval for experimental procedures was granted by the Institutional Animal Care and Research Advisory Committee of KU Leuven.

### Cell isolation and culture

#### Cell isolation

Primary chondrocytes were isolated from growth plates of the proximal tibia and distal femur of 5-day-old male and female mice.^10^ After dissection of the perichondrium, isolated growth plates were incubated with 2 mg·mL^−1^ collagenase type II (Gibco) dissolved in growth medium (GlutaMAX-1 αMEM supplemented with 100 U·mL^−1^ penicillin, 50 µg·mL^−1^ streptomycin and 10% fetal bovine serum (FBS); all from Gibco) for 3 h at 37 °C. The cell suspension was passed through a 70 µm pore cell strainer and centrifuged, and cells were seeded at a density of 7.5 × 10^3^ cells per cm^2^ for experiments.

Periosteum-derived skeletal progenitors were isolated from the long bones of 7- to 9-week-old male mice as described previously.^19^ Briefly, after careful removal of muscle and connective tissue, the epiphyses were embedded in 5% low melting point agarose (Lonza). Next, periosteal progenitor cells were isolated through two consecutive steps of collagenase-dispase digestion (3 mg·mL^−1^ collagenase and 4 mg·mL^−1^ dispase in GlutaMAX-1 αMEM supplemented with 100 U·mL^−1^ penicillin and 50 µg·mL^−1^ streptomycin). The cells collected after the first 10-min digestion were discarded, and the cells collected after the second digestion (50 min) were passed through a 70 μm pore cell strainer. After centrifugation, cells were washed using growth medium and plated at a density of 5 × 10^3^ cells per cm^2^. At 80% confluence, cells were trypsinized and seeded for experiments.

Primary osteoblasts were isolated from the tibiae and femora of 7- to 9-week-old male mice.^9^ Briefly, muscle and connective tissue were thoroughly removed, and bones were incubated in collagenase-dispase digestion solution for 20 min to remove the remaining periosteal cells. Subsequently, the epiphyses were cut off, and the bone marrow was flushed out with PBS. The remaining bone shafts were cut into smaller pieces, and osteoblasts were isolated by collagenase-dispase digestion for 30 min at 37 °C. Finally, the collected cells were passed through a 70 μm pore cell strainer, washed and plated in growth medium at a density of 5 × 10^3^ cells per cm^2^. After three days of culture, RNA was isolated as described below.

#### Nutrient deprivation

Twenty-four hours after cell seeding, chondrocytes were washed with PBS and switched to either control medium, serine/glycine-free medium, glucose deprivation medium or glucose/serine/glycine deprivation medium (Table 1).

**Table 1:** Control and nutrient deprivation media

Medium	Composition
Control	αMEM containing 5 mmol·L^−1^ glucose, 2.5 mmol·L^−1^ glutamine, 0.2 mmol·L^−1^ serine and 0.7 mmol·L^−1^ glycine supplemented with 10% dialyzed FBS, 100 U·mL^−1^ penicillin and 50 µg·mL^−1^ streptomycin
Serine/glycine-free	Serine/glycine-free αMEM containing 5 mmol·L^−1^ glucose and 2.5 mmol·L^−1^ glutamine supplemented with 10% dialyzed FBS, 100 U·mL^−1^ penicillin and 50 µg·mL^−1^ streptomycin
Glucose deprivation	αMEM containing 1 mmol·L^−1^ glucose, 2.5 mmol·L^−1^ glutamine, 0.2 mmol·L^−1^ serine and 0.7 mmol·L^−1^ glycine supplemented with 10% dialyzed FBS, 100 U·mL^−1^ penicillin and 50 µg·mL^−1^ streptomycin
Glucose/serine/glycine deprivation	Serine/glycine-free αMEM containing 1 mmol·L^−1^ glucose and 2.5 mmol·L^−1^ glutamine supplemented with 10% dialyzed FBS, 100 U·mL^−1^ penicillin and 50 µg·mL^−1^ streptomycin

#### In vitro treatments

Unless mentioned otherwise, cells were treated for 72 h with NCT-503 (Sigma-Aldrich, 5 or 10 µmol·L^−1^; DMSO was used as the vehicle control) or supplemented with 1x EmbryoMax Nucleoside Mixture (Merck).

#### Micromass cultures

To analyze matrix deposition by growth plate chondrocytes, we used high-density micromass cultures.^10^ To exclude a potential confounding effect of cell proliferation, chondrocytes were first treated with mitomycin C (50 ng·mL^−1^) for 30 min. Subsequently, 1.5 × 10^5^ cells were resuspended in micromass growth medium (GlutaMAX-1 αMEM supplemented with 1% FBS, 100 U·mL^−1^ penicillin and 50 µg·mL^−1^ streptomycin) and plated as a 10 µL droplet. After cell attachment (1 h at 37 °C), 0.5 mL of micromass growth medium supplemented with 50 µmol·L^−1^ L-ascorbic acid 2-sulfate (Sigma-Aldrich) was added to the wells. After 5 days of culture, micromasses were stained with Alcian Blue. The staining intensity was quantified after Alcian Blue elution (6 mol·L^−1^ guanidine-HCl for 6 h at room temperature), and the absorbance was measured at 620 nm.

#### Growth curve

To analyze cell growth, chondrocytes were seeded at a density of 3 × 10^4^ cells per cm^2^. At the indicated time points, DNA was extracted and quantified by Hoechst staining.

### Genetic targeting

For SOX9 overexpression, skeletal progenitors were transduced with a lentivirus carrying a SOX9 overexpression plasmid (Addgene #36979;^40^ multiplicity of infection (MOI) 150), and for SOX9 knockdown, growth plate chondrocytes were transduced with a lentivirus carrying SOX9 shRNA (Addgene #40645;^40^ MOI 50). For ATF4 knockdown, we used a lentivirus carrying a shRNA against ATF4 (TRCN0000301646, Sigma-Aldrich). All transductions were performed in the presence of 8 μg·mL^−1^ polybrene (Sigma-Aldrich) to enhance the transduction efficiency. Cells were transduced with lentiviruses carrying an empty vector (for SOX9 overexpression) or a scrambled shRNA sequence (for SOX9/ATF4 knockdown) at the same MOI as controls. Virus-containing medium was replaced with normal growth medium after 24 h, and cells were used for further experiments 72 h after transduction.

### Mass spectrometry (MS)-based metabolomics analysis

For metabolic tracing experiments, chondrocytes were cultured in the presence of 5 mmol·L^−1^ ^13^C_6_-glucose (Cambridge Isotope Laboratories) for 72 h. Subsequently, the cells were washed with ice-cold 0.9% NaCl, and metabolites were extracted by harvesting the cells by scraping in 80% methanol supplemented with d27 myristic acid. The samples were analyzed using liquid chromatography-MS as described previously,^10,12^ and metabolite annotation was performed based on an in-house library. Metabolites of interest were analyzed using Xcalibur software (Thermo Fisher Scientific), and the carbon contribution was calculated according to the following equation:

$${{{\mathrm{total}}}}\;{{{\mathrm{carbon}}}}\;{{{\mathrm{contribution}}}} = \frac{{\mathop {\sum }\limits_{i\, =\, 0}^n i * m_i}}{{{{{\mathrm{n}}}} * \mathop {\sum }\limits_{i \,=\, 0}^n m_i}}$$

(*n* denotes the number of carbon atoms in a specific metabolite, *i* refers to the isotopologue, and *m* indicates the abundance of the isotopologue). In-house software was used to correct for naturally occurring isotopologues, and the raw metabolite peak levels were normalized to the protein content. For blood serum metabolite levels, the values were normalized to body weight. Energy homeostasis was assessed by calculating the energy charge based on the normalized raw abundance: [ATP] + ½ [ADP])/([ATP] + [ADP] + [AMP].

### Gene and protein expression analysis

#### Gene expression

RNA was extracted from cells or tissues (NucleoSpin RNA Isolation Kit; Macherey Nagel), and mRNA was reverse transcribed to cDNA (Superscript II Reverse Transcriptase; Thermo Fisher Scientific). Gene expression analysis was performed using specific primer sets (Table 2), and expression levels were calculated using the 2^−ΔΔCt^ method. Values were normalized to *Hprt* expression.

**Table 2:** qRT–PCR primer sequences

Gene	Forward (5′-3′)	Reverse (5′-3′)
*Glut1*	GGGCATGTGCTTCCAGTATGT	ACGAGGAGCACCGTGAAGAT
*Hprt*	TTATCAGACTGAAGAGCTACTGTAATGATC	TTACCAGTGTCAATTATATCTTCAACAATC
*Phgdh*	CGATGAAAGATGGCAAATGGG	GCCACCTCTCTTCCAATTCT
*Psat1*	GCAGCTACTAGACTACAGAGGA	GCTAGCAATTCCCTCACAAGA
*Shmt1*	GGATATCCAGGCCAAAGGTATT	CCAGATGGTAGGCCTGTAATG
*Shmt2*	AGAGGGAGAAGGACAGACAG	CCTCCGAGTACTTGTTGTTGAG
*Mthfd1*	CGCTGACAGATGACGAGATAAA	CCTCAGGAATCTATCGTTGGTG
*Mthfd2*	GTGCTTGGACCAGTACTCTATG	CCAGCCACTACCACATTCTT
*Slc1a4*	CAGTGGACTCTTTCCTCGATTT	ATGGGTCACCACTGTGTAATC
*Slc6a9*	GGGAGGAGCCTTCATGTTC	AGGAAAGCTCCATGAAGAAGAG

#### Protein expression

Total cell lysates and nuclear protein-enriched lysates from cultured cells or ectopic bone implants were obtained using the appropriate cell lysis buffers, and Western blot analysis was performed as described previously.^10,12^ Briefly, proteins were separated by SDS-­PAGE, and protein-containing nitrocellulose membranes were incubated overnight with primary antibodies specific for the following proteins: AMPK (#2532; Cell Signaling Technology), p-AMPK^T172^ (#2535, Cell Signaling Technology), ATF4 (#11815, Cell Signaling Technology), β-­actin (A5441, Sigma-Aldrich), eIF2α (#9722, Cell Signaling Technology), p-eIF2α^S51^ (#9721, Cell Signaling Technology), Lamin A/C (sc-376248, Santa Cruz Biotechnologies), PHGDH (#66350, Cell Signaling Technology) and PSAT1 (NBP1-55368, Bio-Techne). Appropriate HRP-conjugated secondary antibodies were used for chemiluminescent detection of proteins (Western Lightning Plus, PerkinElmer).

### Proliferation assay

Chondrocyte proliferation was evaluated using the Cell Proliferation Biotrack ELISA system (GE Healthcare) according to the manufacturer’s instructions. Briefly, 72 h after cell seeding, 5′-bromo-2′-deoxyuridine (BrdU) was added 4 h prior to measurement, and the obtained values were normalized to the DNA content.

### Flow cytometric analyses

#### Cell viability

Cell viability was analyzed by flow cytometry (Gallios flow cytometer, Beckman Coulter) using FITC-labeled Annexin V and propidium iodide (PI) (Dead Cell Apoptosis Kit, Thermo Fisher Scientific) directly after chondrocyte isolation or 72 h after cell seeding. The number of viable cells (Annexin V^-^PI^-^) was determined using Flowing software (University of Turku, Finland).

#### Protein synthesis

Protein synthesis in cultured chondrocytes was analyzed using a Click-iT L-Homopropargylglycine (HPG) Alexa Fluor 488 Protein Synthesis Assay Kit (Thermo Fisher Scientific) 72 h after cell seeding, as described previously.^10^ The mean fluorescence intensity of HPG-Alexa Fluor 488 was calculated using Flowing software.

#### Reactive oxygen species (ROS) levels

ROS levels in cultured chondrocytes were measured using the fluorescent probe CM-H_2_DCFDA (Thermo Fisher Scientific) 72 h after cell seeding. CM-H_2_DCFDA (5 µmol·L^−1^) was added to the culture medium for 30 min, and fluorescence was detected by flow cytometry. The mean fluorescence intensity of CM-H_2_DCFDA was calculated using Flowing software.

### Collagen synthesis

Collagen synthesis, analyzed 72 h after cell seeding, was quantified as described previously.^12^ Briefly, chondrocytes were incubated overnight with 20 µCi per mL ^3^H-proline (PerkinElmer) and subsequently lysed in 11% acetic acid-H_2_O supplemented with 0.25% bovine serum albumin. Proteins were precipitated by the addition of 20% trichloroacetic acid, and the pellet was resuspended in scintillation fluid. Finally, radioactivity was determined by liquid scintillation counting, and the values were normalized to the DNA content.

### Ectopic bone ossicle model

As an alternative in vivo model of endochondral ossification, we used a validated ectopic bone ossicle model.^10,12,28^ Briefly, periosteal skeletal progenitors isolated from wild-type C57BL/6 or *Phgdh*^*fl/fl*^ mice (see above) were cultured in growth medium supplemented with 5 ng·mL^−1^ human recombinant fibroblast growth factor 2 (FGF2) (R&D Systems) and 5 U·mL^−1^ heparin (LEO Pharma). After expansion, FGF2-pretreated cells were encapsulated in a type I collagen gel (5 mg·mL^−1^ in PBS, Corning GmbH) at a density of 1 × 10^7^ cells per mL and injected subcutaneously. Ectopic implants were collected one week after implantation, when the cartilaginous matrix was formed,^28^ and either fixed with 2% paraformaldehyde and processed for histological analysis or used for RNA and protein extraction. Ossicle volume (*V*) was calculated based on the following equation:

$$V = \frac{{W^2 \times L}}{2}$$

where *L* is the ossicle length and *W* is the ossicle width.

To obtain mice with PHGDH deletion, FGF2-pretreated *Phgdh*^*fl/fl*^ periosteal cells were transduced prior to implantation with a Cre recombinase-expressing adenovirus (MOI 500; Gene Transfer Vector Core, University of Iowa, USA). As a control, *Phgdh*^*fl/fl*^ periosteal cells were transduced with an adenovirus carrying an empty vector (Gene Transfer Vector Core). The virus-containing medium was changed to heparin/FGF2-containing culture medium after 24 h, and cells were implanted 72 h after transduction.

### Semistabilized fracture model

The tibial semistabilized fracture model was established in 8-week-old male C57BL/6 mice as previously described.^10,41^ For PHGDH inhibition, mice were injected with 50 µL of a NCT-503 preparation (40 μg per g body weight) locally at the fracture site daily, starting 3 days after fracture induction. NCT-503 was dissolved in a vehicle composed of ethanol (5%), PEG 300 (35%), and a 30% (w/v) hydroxypropyl-β-cyclodextrin solution (60%). All chemicals were obtained from Sigma-Aldrich. Ten days after fracture induction, tibiae were collected, fixed overnight with 2% paraformaldehyde and subsequently processed for histology.

### Serine/glycine-free diet

In the ectopic ossicle implantation and long bone fracture experiments, wild-type mice were fed either an amino acid control diet (TD.110839; Envigo) or a serine/glycine-free diet (TD.160752; Envigo) starting 3 days before the surgical procedure. The diet formulations are listed in.^42^ The serine/glycine-free diet contained more corn starch (407.88 g·kg^−1^) than the amino acid control diet (381.18 g·kg^−1^) to compensate for the loss of serine and glycine. All other nutrients were the same.

### X-ray microtomography

Mineralized bone tissue was analyzed using X-ray microtomography (microCT) as described previously.^43^ Using the SkyScan 1172 microCT system (Bruker), tibiae were scanned with a 50 kV tube voltage, 200 µA current and 0.5 mm aluminum filter to produce a 5 µm pixel size. NRecon software (Bruker) was used for projection data reconstruction, and trabecular and cortical volumes of interest were manually selected. 3D bone morphometric parameters were calculated using CT Analyzer software (Bruker) in accordance with the American Society for Bone and Mineral Research guidelines.^44^

### Histochemistry and histomorphometry

Histomorphometric analyses were performed as previously described.^10^ Quantification of growth plate length was performed on H&E-stained sections. To account for changes in growth plate morphology, the lengths of the proliferative and hypertrophic zones, as well as the total growth plate length, were measured at five different sites equally distributed across the width of the growth plate. Safranin O staining was used to visualize cartilage matrix proteoglycans.

For immunohistochemical staining,^10^ tissue sections were incubated overnight with primary antibodies against BrdU (proliferating cells; OBT0030, Bio–Rad), COL2 (cartilage; MAB8887, Chemicon) and PHGDH (#66350, Cell Signaling Technology). For BrdU immunostaining, mice were injected intraperitoneally with 150 µg·g^−1^ BrdU in saline solution 4 h before sacrifice. Before incubation with the primary anti-COL2 antibody, sections were predigested (0.025% pepsin in 0.2 mol·L^−1^ HCl, 10 minutes at 37 °C), fixed (4% paraformaldehyde), permeabilized (0.2% Triton X-100) and quenched (50 mmol·L^−1^ NH_4_Cl). Appropriate fluorophore-labeled secondary antibodies were used for signal visualization, and sections were stained with Hoechst to visualize cell nuclei.

After image acquisition (Axioplan 2; Zeiss, Belgium), histomorphometric analysis was performed using the related AxioVision software (Zeiss). All staining was quantified in the total growth plate area unless otherwise specified. Cell density in bone ossicles was quantified in Hoechst-stained sections.

### Statistics

Data are presented as the mean ± standard deviation values. In all figures, *n* represents the number of independent experiments (for each in vitro experiment, at least three technical replicates were used) or the number of individually phenotyped mice/ossicles. For immunoblot analyses, at least three independent experiments were performed, and representative images are shown. Statistical analysis (GraphPad Prism 9 software) was performed using unpaired two-tailed Student’s *t* test or one-way/two-way ANOVA followed by the Tukey–Kramer post hoc test, as specified in the figure legends. *P* values of <0.05 were considered statistically significant.

### Figure artwork

Figure artwork was created with Biorender.com.

## Supplementary information

Supplementary information
