# Supplementary material for: De novo serine synthesis regulates chondrocyte proliferation during bone development and repair
Source: Bone Res. 2022 Feb 15;10:14. doi: 10.1038/s41413-021-00185-7 (PMC8844408; doi:10.1038/s41413-021-00185-7)
Supplement: Supplementary file 1 — Supplementary information [file 41413_2021_185_MOESM1_ESM.docx]

**Supplementary information**

Supplementary figures 1-6

***De novo* serine synthesis regulates chondrocyte proliferation during endochondral ossification**

Steve Stegen^a^, Shauni Loopmans^a^, Ingrid Stockmans^a^, Karen Moermans^a^, Peter Carmeliet^b-d^ and Geert Carmeliet^a^

**Supplementary figure 1**

**
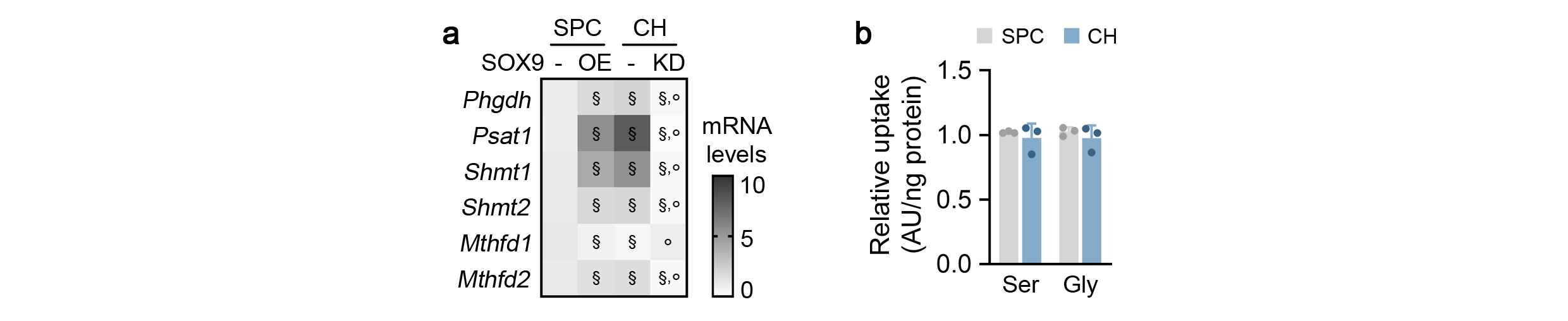
**

**Supplementary figure 1. Active *de novo* serine synthesis in growth plate chondrocytes**

**a** *Phgdh*, *Psat1*, *Shmt1*, *Shmt2*, *Mthfd1* and *Mthfd2* mRNA levels in cultured skeletal progenitor cells (SPC) and growth plate chondrocytes (CH) after transduction with a lentiviral vector carrying a SOX9-overexpression (OE) plasmid or a shRNA (KD) against SOX9, respectively (n=3). An empty vector or scrambled shRNA (shScr; -) were used as respective controls.

**b** Relative serine (Ser) and glycine (Gly) uptake in SPC and CH (n=3).

Data are means ± SD, ^§^p<0.05 vs shScr-SPC, °p<0.05 vs shScr-CH (ANOVA).

**Supplementary figure 2**


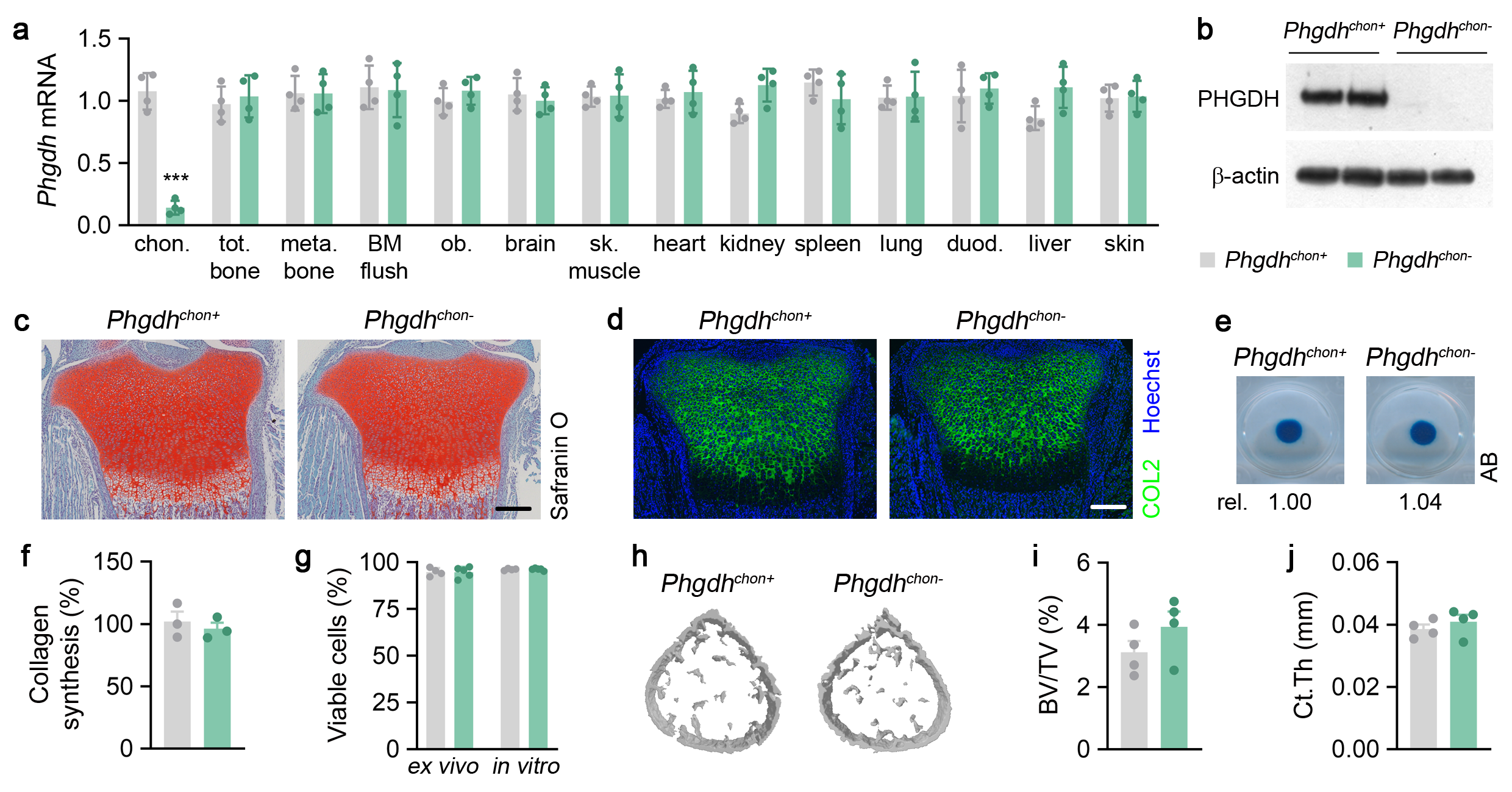


**Supplementary figure 2. PHGDH deletion in chondrocytes using *Col2-Cre* transgenic mice**

**a** *Phgdh* mRNA levels in chondrocytes (chon.), total neonatal bone (tot. bone), metaphyseal juvenile bone (meta. bone), cells obtained after bone marrow (BM) flush, trabecular osteoblasts (ob.), brain, skeletal (sk.) muscle, heart, kidney, spleen, lung, duodenum (duod.), liver and skin isolated from wild-type (*Phgdh^chon+^*) and chondrocyte-specific PHGDH knockout (*Phgdh^chon-^*) mice (n=4).

**b** Immunoblot of PHGDH and β-actin levels on growth plate tissue isolated from *Phgdh^chon+^* and *Phgdh^chon-^* mice (n=4-5). Two biological replicates are shown.

**c-d** Safranin O staining (**c**) and COL2 immunostaining (**d**) of neonatal growth plates from *Phgdh^chon+^* and *Phgdh^chon-^* mice (n=4-5). Scale bars are 200 µm.

**e** Matrix deposition by wild-type and PHGDH-deficient chondrocytes cultured as micromasses, as determined by Alcian Blue (AB) staining (n=3).

**f** Collagen synthesis by wild-type and PHGDH-deficient chondrocytes (n=3).

**g** Cell viability of wild-type and PHGDH-deficient chondrocytes, as evidenced by Annexin V (AnxV) - propidium iodide (PI) flow cytometry, directly after isolation (*ex vivo*) or after 3-day culture (*in vitro*) (n=4-5). AnxV^-^PI^-^ cells were considered viable.

**h-j** 3D microCT image of the metaphyseal region of neonatal long bones from *Phgdh^chon+^* and *Phgdh^chon-^* mice (**h**), with quantification of trabecular bone volume (**i**; BV/TV) and cortical thickness (**j**; Ct.Th).

Data are means ± SD, ***p<0.001 vs *Phgdh^chon+^* (Student’s *t*-test).

**Supplementary figure 3**


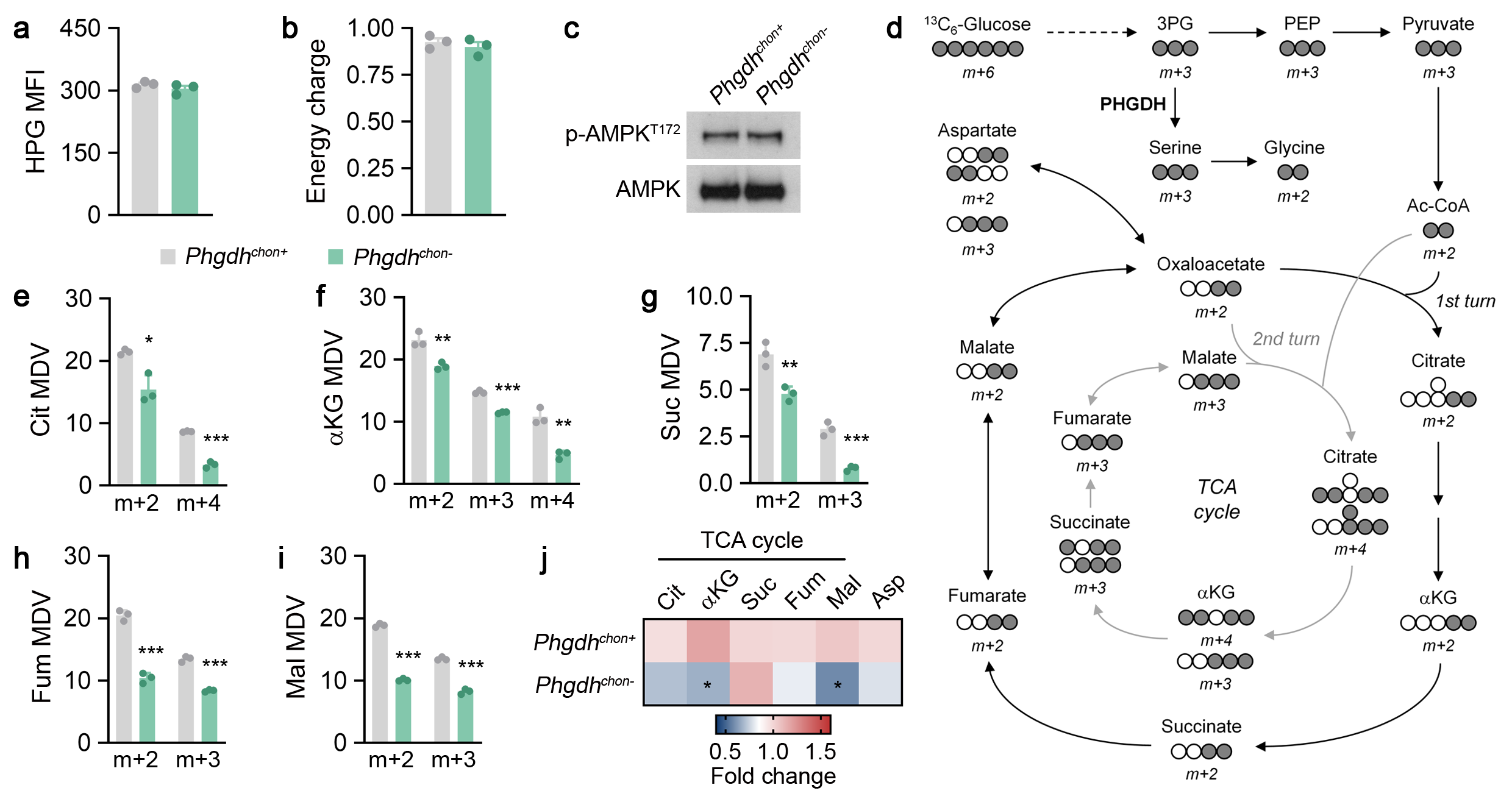


**Supplementary figure 3. Effect of PHGDH deletion on biosynthetic and bioenergetic pathways**

**a** Protein synthesis, as determined by L-homopropargylglycine (HPG) flow cytometry, by chondrocytes from wild-type (*Phgdh^chon+^*) and chondrocyte-specific PHGDH knockout (*Phgdh^chon-^*) mice (n=3).

**b** Energy charge, calculated as [ATP] + ½ [ADP] / [ATP] + [ADP] + [AMP], in wild-type and PHGDH-deficient chondrocytes (n=3).

**c** Immunoblot of phosphorylated AMPK (Threonine 172; p-AMPK^T172^) and AMPK levels in wild-type and PHGDH-deficient chondrocytes (n=3).

**d** Schematic of carbon atom (circles) transitions of ^13^C_6_-glucose used to detect label incorporation in depicted metabolites. 3PG is 3-phosphoglycerate, PEP is phosphoenolpyruvate, Ac-CoA is acetyl-CoA, αKG is α-ketoglutarate.

**e-i** Citrate (Cit; **e**), αKG (**f**), succinate (Suc; **g**), fumarate (Fum; **h**) and malate (Mal; **i**) labeling from ^13^C_6_-glucose in wild-type and PHGDH-deficient chondrocytes (n=3). Specific mass distribution vectors (MDVs) for each metabolite are shown.

**j** Intracellular Cit, aKG, Suc, Fum, Mal and aspartate (Asp) levels in wild-type and PHGDH-deficient chondrocytes (n=3).

Data are means ± SD, *p<0.05 vs *Phgdh^chon+^*, **p<0.01 vs *Phgdh^chon+^*, ***p<0.001 vs *Phgdh^chon+^* (Student’s *t*-test).

**Supplementary figure 4**


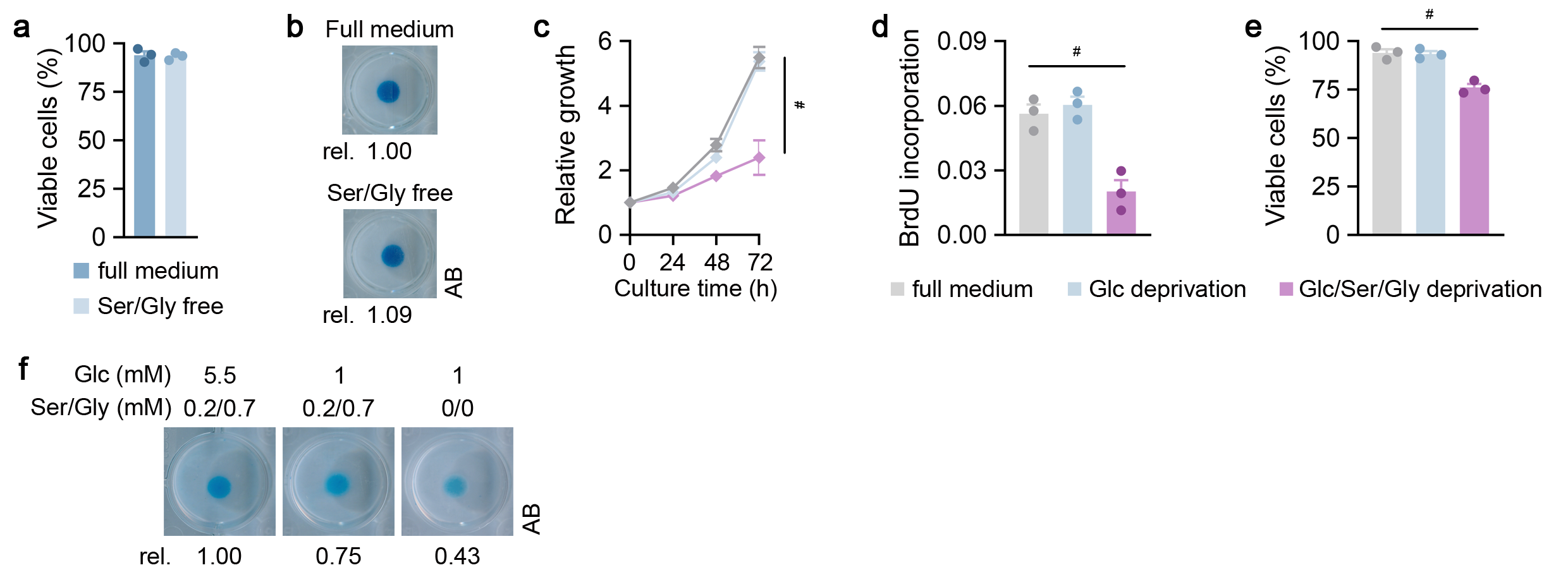


**Supplementary figure 4. Additional glucose deprivation sensitizes chondrocytes to serine/glycine starvation**

**a-b** Cell viability (**a**; quantified by AnxV-PI flow cytometry, viable cells are AnxV^-^PI^-^) and matrix deposition (**b**; evidenced by Alcian Blue (AB) staining) by chondrocytes cultured in full medium or serine/glycine (Ser/Gly) free medium (n=3).

**c** Growth curve, analyzed by DNA quantification, of chondrocytes cultured in full medium or in low-glucose (Glc) containing medium (1 mM Glc), with or without serine/glycine starvation (0 mM Ser, 0 mM Gly) (n=3).

**d-f** Proliferation (**d**; quantified by BrdU incorporation), viability (**e**; quantified by AnxV-PI flow cytometry) and matrix deposition (**f**; evidenced by AB staining) by chondrocytes cultured in full medium or in low-glucose containing medium, with or without serine/glycine starvation (n=3).

Data are means ± SD, ^#^p<0.05 (ANOVA).

**Supplementary figure 5**


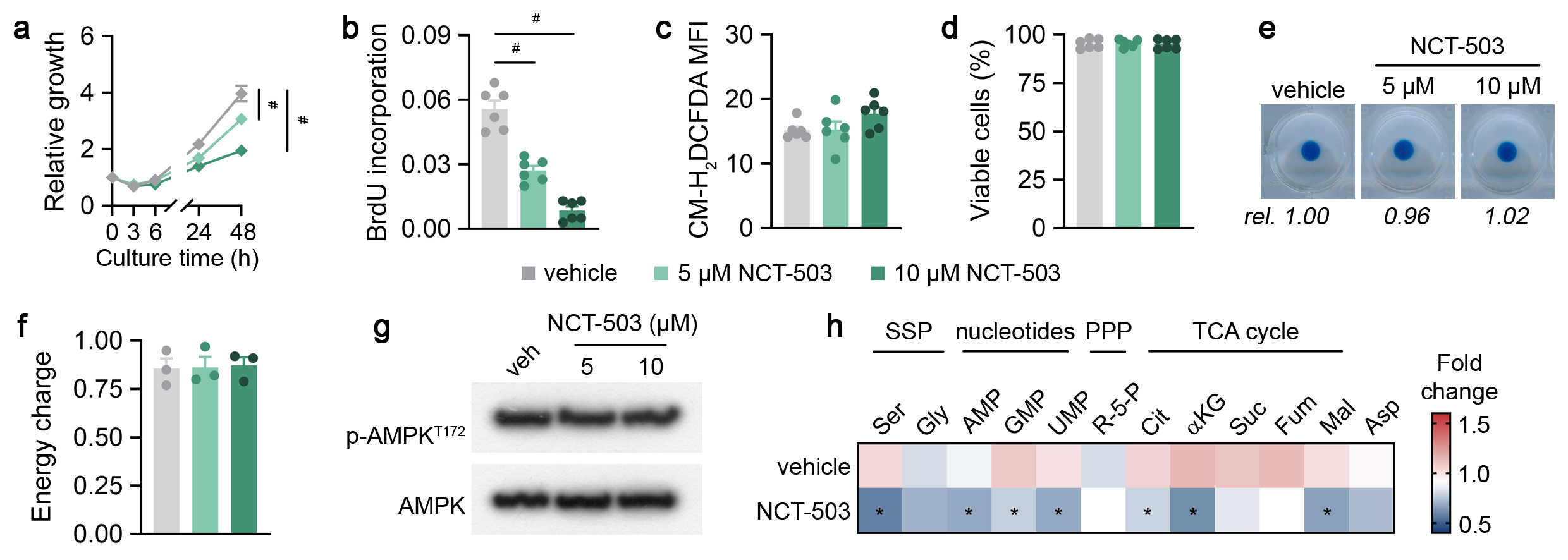


**Supplementary figure 5. Pharmacological PHGDH inhibition mirrors genetic PHGDH inactivation**

**a** Growth curve of chondrocytes treated with two different concentrations of NCT-503 (n=6).

**b-e** Proliferation (**b**; quantified by BrdU incorporation), ROS levels (**c**; quantified by CM-H_2_DCFDA flow cytometry), viability (**d**; quantified by AnxV-PI flow cytometry, AnxV^-^PI^-^ are viable cells) and matrix deposition (**e**; evidenced by Alcian Blue (AB) staining) of chondrocytes treated with different concentrations of NCT-503 (n=6).

**f** Energy charge, calculated as [ATP] + ½ [ADP] / [ATP] + [ADP] + [AMP], in chondrocytes treated with different concentrations of NCT-503 (n=3).

**g** Immunoblot of phosphorylated AMPK (Threonine 172; p-AMPK^T172^) and AMPK levels in chondrocytes treated with different concentrations of NCT-503 (n=3).

**h** Intracellular levels of depicted metabolites in chondrocytes treated with 5 µM NCT-503 (n=3). SSP is serine synthesis pathway, PPP is pentose phosphate pathway, Ser is serine, Gly is glycine, R-5-P is ribose-5-phosphate, Cit is citrate, αKG is α-ketoglutarate, Suc is succinate, Fum is fumarate, Mal is malate and Asp is aspartate.

Data are means ± SD, ^#^p<0.05 (ANOVA), *p<0.05 vs vehicle (Student’s *t*-test).

**Supplementary figure 6**


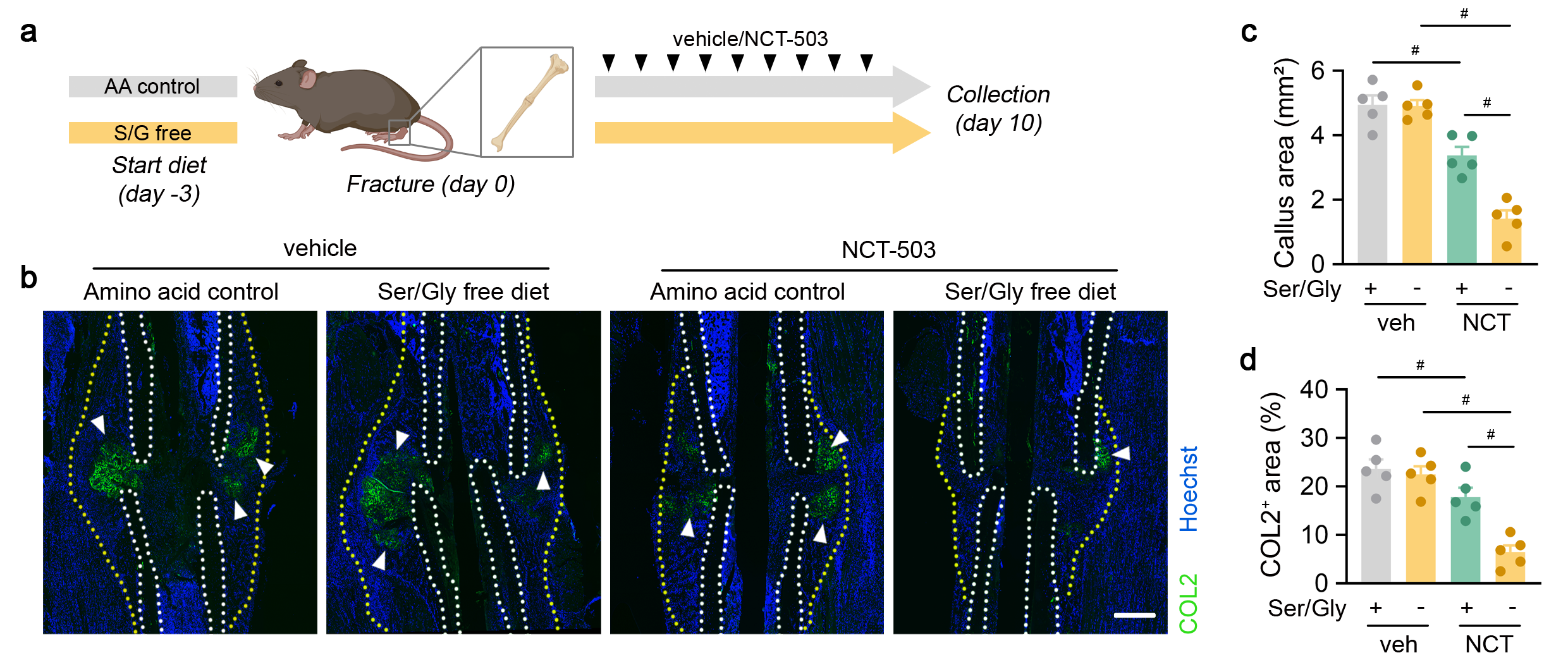


**Supplementary figure 6. Combined PHGDH inhibition and serine/glycine starvation impairs endochondral bone healing**

**a** Schematic overview of the experimental setup. Tibial fractures were induced in mice fed an amino acid (AA) control or serine/glycine-free diet. Mice were injected locally with vehicle or NCT-503 in the fracture callus every day for a total of 10 days.

**b** COL2 immunostaining at post-fracture day 10 of vehicle and NCT-503 treated mice (n=5). Scale bar is 250 µm, white arrowheads indicate COL2-positive cartilage, white dotted lines indicate fractured bone, yellow dotted lines indicate fracture callus.

**c-d** Quantification of total callus area (**c**) and COL2-positive area in the callus (**d**) at post-fracture day 10 of vehicle and NCT-503 treated mice (n=5).

Data are means ± SD, ^#^p<0.05 (ANOVA).
